# QBMG: quasi-biogenic molecule generator with deep recurrent neural network

**DOI:** 10.1186/s13321-019-0328-9

**Published:** 2019-01-17

**Authors:** Shuangjia Zheng, Xin Yan, Qiong Gu, Yuedong Yang, Yunfei Du, Yutong Lu, Jun Xu

**Affiliations:** 1 Research Center for Drug Discovery, School of Pharmaceutical Sciences, Sun Yat-Sen University, 132 East Circle at University City, Guangzhou, 510006 China; 2 School of Computer Science and Technology, Wuyi University, 99 Yingbin Road, Jiangmen, 529020 China; 3 National Supercomputer Center in Guangzhou and School of Data and Computer Science, Sun Yat-Sen University, 132 East Circle at University City, Guangzhou, 510006 China

**Keywords:** Deep learning, Recurrent neural networks, Natural product, Virtual library

## Abstract

Biogenic compounds are important materials for drug discovery and chemical biology. In this work, we report a quasi-biogenic molecule generator (QBMG) to compose virtual quasi-biogenic compound libraries by means of gated recurrent unit recurrent neural networks. The library includes stereo-chemical properties, which are crucial features of natural products. QMBG can reproduce the property distribution of the underlying training set, while being able to generate realistic, novel molecules outside of the training set. Furthermore, these compounds are associated with known bioactivities. A focused compound library based on a given chemotype/scaffold can also be generated by this approach combining transfer learning technology. This approach can be used to generate virtual compound libraries for pharmaceutical lead identification and optimization.

**Electronic supplementary material:**

The online version of this article (10.1186/s13321-019-0328-9) contains supplementary material, which is available to authorized users.

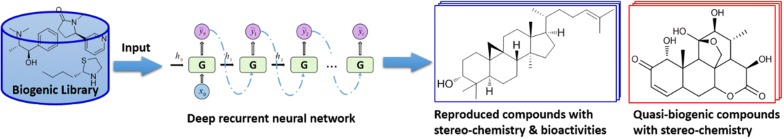

## Introduction

Biogenic compounds are important for medicinal chemistry and chemical biology [1]. It was reported that more than 50% of marketed drugs were derived from biogenic molecules [2]. The reason is that both biogenic compounds and pharmaceutical agents are biologically relevant and recognized by organisms [3]. However, it requires tremendous efforts to identify and isolate biogenic compounds from natural resources [4]. Current virtual screening technologies allow us to efficiently identify biogenic molecules for pharmaceutical uses [5], but, it is getting rare to identify biogenic compounds with new scaffolds [6]. Practically, biogenic compounds can be probes for pharmaceutical and biological studies and, inspire chemists to make quasi-biogenic compounds (compounds modified from natural products).

Hence, many experimental approaches have been reported to synthesize quasi-biogenic compound libraries, such as targeted-oriented synthesis [7, 8], diversity-oriented synthesis (DOS) [7, 9, 10], biology-oriented synthesis (BIOS) [11, 12], and functional-oriented synthesis (FOS) [13]. Meanwhile, virtual quasi-biogenic compound library generation methods were also reported, such as Yu reported a recursive atom-based compound library enumeration [14]. Based on property distribution analyses on the differences between drugs, natural products, and combinatorial libraries, Feher and Schmidt reported that those natural product-like libraries were more like synthetic compounds rather than natural products [15]. The entirety of the biologically relevant chemical space is largely ignored [1]. Therefore, the biological relevant features should be taken into account while generating natural product-like libraries.

Recent advances in deep learning technology have brought many achievements in small molecule and peptide design. Aspuru-Guzik’s group reported an automated chemical design using a data-driven continuous representation of molecules [16]. Segler and colleagues reported a method to generate virtual focused library using recurrent neural networks fine-tuned with small number of known active compounds [17]. Later, more studies were done by combining deep reinforcement learning [18], Monte Carlo search [19], de novo peptide design method [20], and generative adversarial network [21].

The main deficits of previous approaches are (1) stereochemistry was not explicitly considered in the generated libraries, (2) there was no de novo approach to generate focused libraries biased on a specified scaffold/chemotype (this is important for lead optimization in medicinal chemistry). In the meantime, we are not aware of any models that used to construct specific virtual biogenic-like compounds libraries of the type we envisioned.

In this work, we report a deep recurrent neural networks (RNN) [22] with gate recurrent unit (GRU) [23] to overcome these deficits, and generate quasi-biogenic compound library to explore greater biogenic diversity space for medicinal chemistry and chemical biology studies. By combining transfer learning [24], we can build focused compound libraries biased on a specific chemotype for lead optimization.

## Methods

### Biogenic compound structure data

163,000 biogenic compound structures were derived from biogenic library of ZINC15 [25]. These compounds are primary and secondary metabolites. The chemical structure data were converted in canonicalized SMILES format [26]. The chemical structures were filtered by removing the molecules containing metal elements, small molecules (the number of non-hydrogen atoms less than 10), and larger molecules (the number of non-hydrogen atoms greater than 100). This process resulted in 153,733 biogenic structures.

### ZINC biogenic-like compound reference

5060 ZINC biogenic-like compounds were collected from biogenic-like subset of ZINC12 [27]. This library consisted of synthetic compounds that having Tanimoto 80% similarity or better with biogenic library.

### Active compound reference

The compounds in ChEMBL23 [28] are used as active compound references.

### Molecular representation and tokenization

**Fig. 1:**
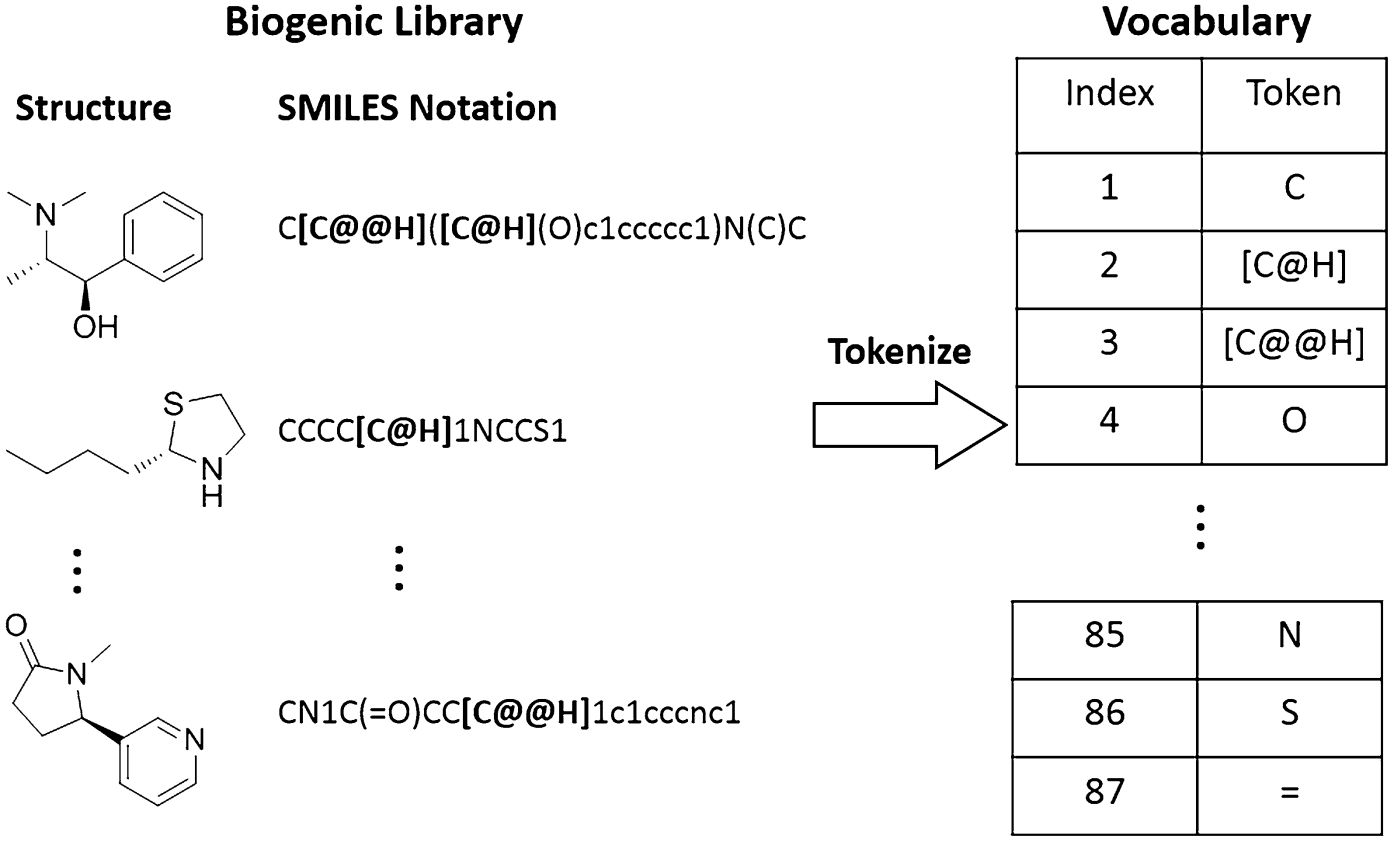
Molecular representation and tokenization. The SMILES notations grouped by a pair of square brackets is considered as one token

Biogenic molecules have many chiral centers, charges, cyclic connection descriptive SMILES notations, which are called as tokens in machine learning studies, such as @, = , #, etc. Conventionally, each letter in a molecular SMILES notation was sent to RNNs for training. This process cannot reflect the biogenic features of chiral centers, charges, cyclic connection descriptors. To preserve these important features, we train RNNs with normal tokens and combined tokens (the SMILES notations grouped by a pair of square brackets []). With this rule, the original SMILES data consisted the vocabulary as shown in Fig. 1. Compared to 35 tokens and average sequence length of 82.1 ± 34.9 (mean ± SD) with conventional method, this way resulted in 87 tokens and average sequence length of 62.4 ± 25.27 in biogenic library.

### Word embedding process

In a conventional one-hot encoding approach, each molecule is represented by a number of token vectors. All token vectors have the same length (in our case, it is 87 as shown in Fig. 1). Each component in a vector is set as zero except the one at the token’s index position. This data storage protocol occupies great memory space and result in inefficient performance. Therefore, we adopt word embedding, which is usually used in natural language process [29]. With this method, each conventional token vector was compressed into an information enriched vector. Thus, a token transformed from a space with one dimension per word to a continuous vector through unsupervised learning. This data representation can record the “semantic similarity” of every token. This process expedites the convergence of a training [30]. In summary, each molecular structure in our work is converted in a SMILES string, which is then encoded into a one-hot matrix, and then is transformed to a word embedding matrix at the embedding layer.

### Network architecture

**Fig. 2:**
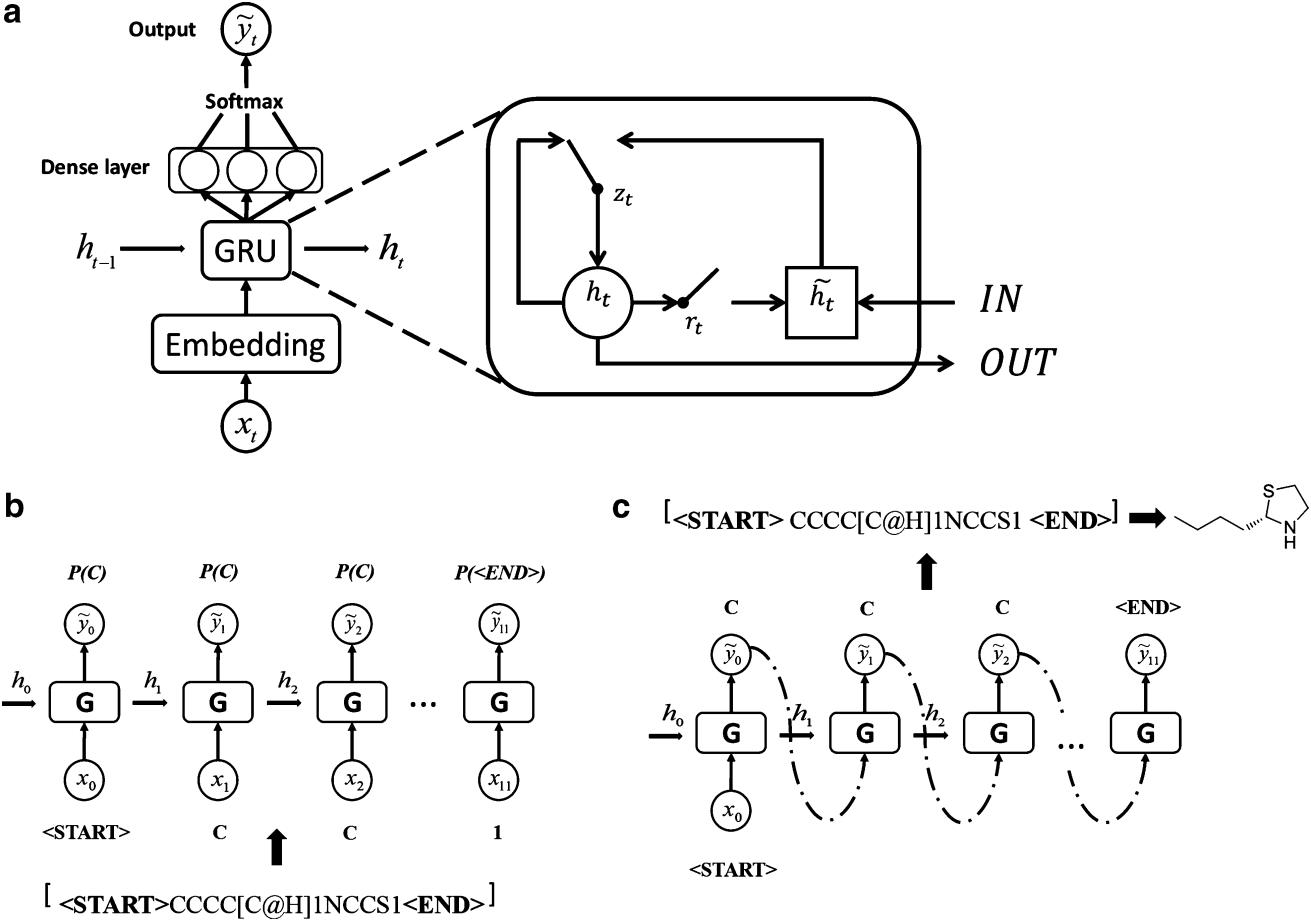
Network architecture and training procedure. **a** Unfolded representation of the training model, which contains embedding layer, GRU structure, fully-connected linear layer and output layer. The structure of GRU cell is detailed on the right. **b** Flow-chart for the training procedure with a molecule. A vectorized token of the molecule is input as $x_{t}$ in a time step, and the probability of the output to $x_{t + 1}$ as the next token is maximized. **c** The new molecular structure is composed by sequentially cascading the SMILES sub-strings replied by the RNN network

The modified recurrent neural network structure is depicted in Fig. 2a. The whole model consists of embedding layer, GRU structure and densely connected layer. The embedding layer consists of 64 units, which translate every single token from a one-hot vector to a 64-dimensional vector. This vector is then transferred to GRUs. The GRUs consists 3 layers, in which each layer has 512 neurons. The GRU output data to the densely connected linear layer with 89 neurons, combining the output signals with a *softmax* function. The number of the neurons in densely connected layer is the same as the number of the vocabularies. <START> and <END> are additional tokens, which mark the starting and ending of a SMILES string. For a GRU cell (Fig. 2a), $h_{t }$ is the hidden state and $\tilde{h}_{t}$ is the candidate hidden state.$r_{t }$ and $z_{t }$ are reset gate and update gate. With these gates, the network ‘knows’ how to combine the new input with the previously memorized data and update the memory. The details of GRU operations are described in Additional file 1.

### Training procedure

Training an RNN for generating SMILES strings is done by maximizing the probability of the next token positioned in the target SMILES string based on the previous training steps. At each step, the RNN model produces a probability distribution over what the next character is likely to be, and the aim is to minimize the loss function value and maximize the likelihood assigned to the expected token. The parameters $\theta$ in the network were trained with following loss function $J\left( \theta \right)$:

$$J\left( \theta \right) = - \mathop \sum \limits_{t = 1}^{T} \log P(x^{t} |x^{t - 1} , \ldots ,x^{1} )$$

Simplified depiction of the training procedure with one biogenic molecule has been shown in Fig. 2b.

### Sampling procedure

The model predicts biogenic molecules based upon the token probability distributions learned from the training set. The prediction consists of the following steps:

1. a <START> token is sent to the network;
2. the network replies with another token (a SMILES sub-string);
3. the new token is sent to the network again to get a newer token;
4. repeat (3) till the network replies with <END> token.

The new molecular structure is composed by sequentially cascading the SMILES sub-strings replied by the RNN network.

The GRU model was implemented using Pytorch library [31], and trained with ADAM optimizer [32] using a batch size of 128 and 0.001 learning rate. The model was trained until convergence. For each training epoch, a sampled set of 512 SMILES strings was generated to evaluate the validity using RDkit [33].

### Validating the predicted compound library

The following criteria are used to evaluate the compound library generated by the RNN model.

1. *Natural product*-*likeness.* Natural product-likeness score [34], a Bayesian measure which allows for the determination of how molecules are similar to the chemical space covered by natural products based on atom-center fragment (a kind of fingerprint), were implemented to score the generated molecules. Note that we used the version that was packaged into RDkit in 2015.
2. *Physico*-*chemical properties/descriptors*. To visually compare the generated library against the biogenic library (the training set) and ZINC biogenic-like library, t-SNE (t-distributed stochastic neighbor embedding) maps are calculated with a set of physico-chemical properties/descriptors (cLogP, MW, HDs, HAs, rotatable bonds, number of aromatic ring systems, and TPSA) following the method reported by Chevillard and Kolb [35]. It is believed that biogenic compounds are structurally diverse in terms of molecular weight, polarity, hydrophobicity, and aromaticity [3, 36, 37].
3. *Ability to reproduce biogenic molecules*. The generated compound library should be able to reproduced already existed biogenic molecular structures [17]. To validate the ability to reproduce biogenic molecules, a variant five-fold cross-validation method is used. The process consisted of the following steps:
4. the biogenic library was randomly divided into five sub-libraries (each sub-library has 30,747 compounds);
5. these sub-libraries were used in a five-fold cross-validation protocol (one sub-library was used as the test set; the others were used as the training sets) to validate the RNN model;
6. sampling 153,733 (the same number of compounds in the biogenic library) unique compounds excluding the repeated ones in the training sets each fold after training;
7. comparing the generated library against the test library to identify overlapped molecules, and calculate the ratio of reproduced compounds;
8. the five-fold cross-validation process was repeated for three times.
9. *Scaffold validation.* To validate the new scaffold generation capacity of the RNN model, the generated, training and test libraries were analyzed using scaffold-based classification (SCA) method [38]. The Tanimoto similarities of the scaffolds derived from the generated library and training library were calculated with standard RDKit similarity based on ECFP6 molecular fingerprints [39]. These similarities were used to compare the generated new scaffolds against the biogenic scaffolds.

### Transfer learning for chemotype-biased library generation

It is important to generate a chemotype-biased library for lead optimization if a privileged scaffold is known. The transfer learning process consists of the following steps:

1. selecting focused compound library (FCL) from the biogenic library. All compounds in FCL have a common scaffold/chemotype;
2. re-trained the RNN model with FCL;
3. predict a chemotype-biased library.

## Results and discussion

The ZINC biogenic library with 153,733 compounds were used to train an RNN model. Along with the number of the epochs grew, the model was converging (See Additional file 2 for learning curves). After training for 50 epochs, the model can generate an average of 97% valid SMILES strings. 250,000 valid and unique SMILES strings were generated as the predicted library. After removing compounds that were found in the training set from the predicted library, we got 194,489 compounds. The average number of tokens for each compound was 59.4 ± 23.1 (similar to the one for a compound in the biogenic library). 153,733 (the same number of the compounds in the training library) compounds were selected from the predicted library to study their natural product-likeness and physico-chemical properties/descriptor profiles.

### Natural product-likeness of the predicted library

**Fig. 3:**
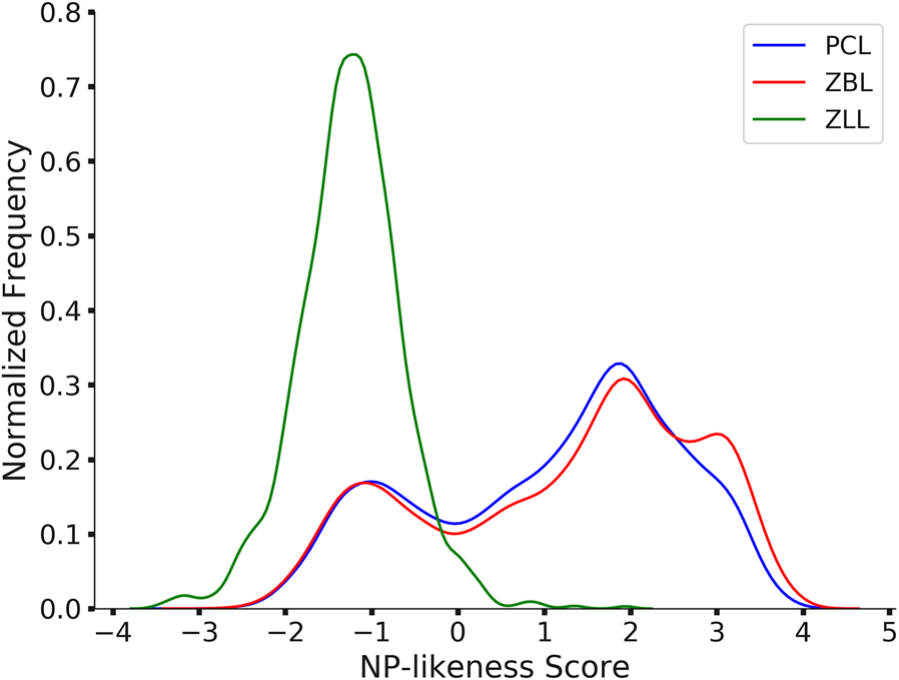
PCL is quasi-biogenic and ZBL is biogenic, and they are similar to each other. ZLL is different from ZBL, and only partially overlapping the biogenic chemical diversity space

The natural product-likenesses of ZINC biogenic library (ZBL), ZINC biogenic-like library (ZLL), and our predicted compound library (PCL) were compared as shown in Fig. 3. The average natural product-likeness scores of PCL and ZBL were 1.09 ± 1.46 (mean ± SD) and 1.22 ± 1.56, indicating that they were both natural product-like, and similar to each other. The average natural product-likeness of a ZLL compound was − 1.25 ± 0.60, indicating that ZLL compounds were different from ZBL compounds, and compounds only partially overlapped the biogenic chemical diversity space.

**Fig. 4:**
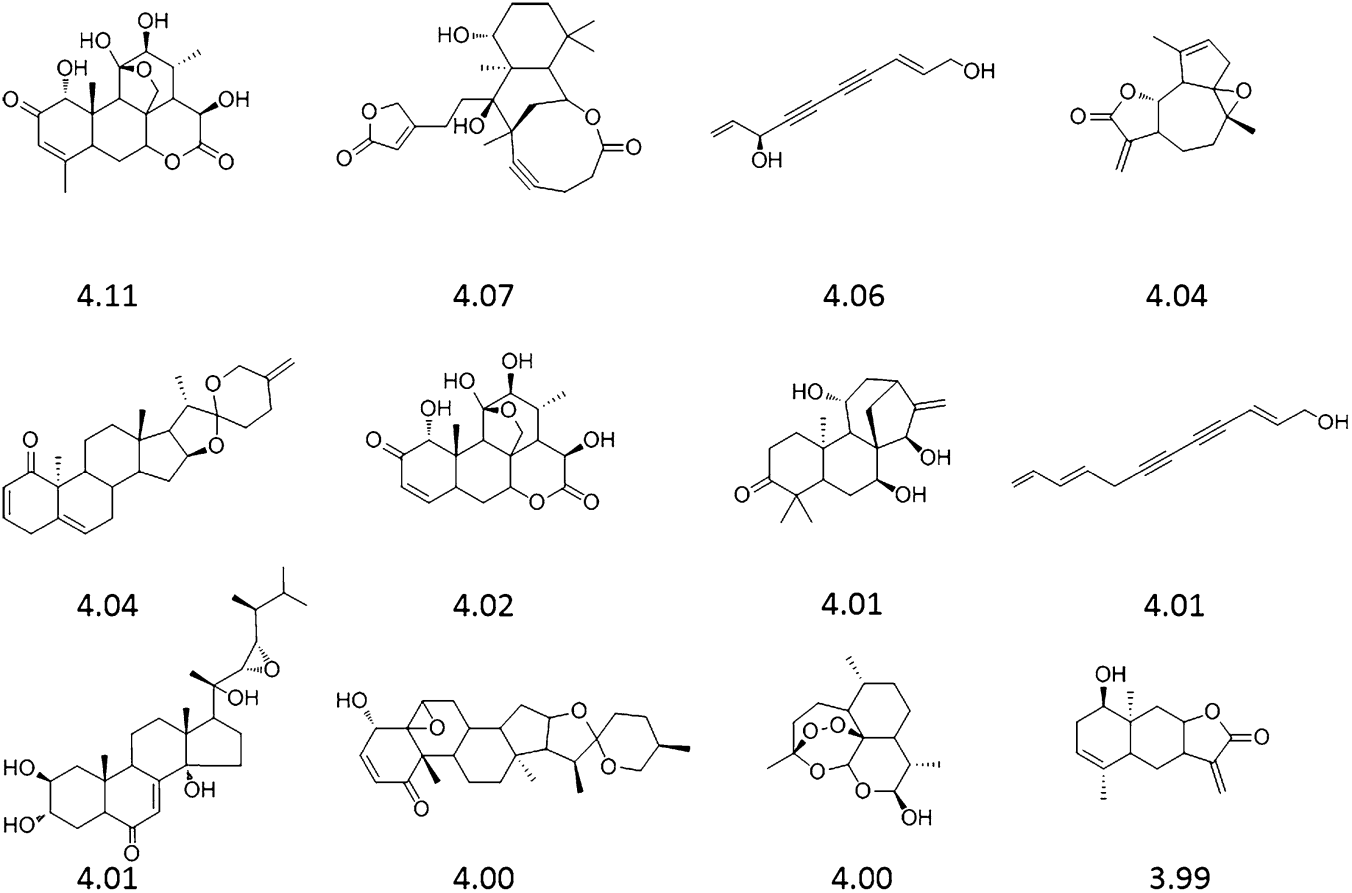
Top-twelve PCL compounds and their natural product-likeness scores

The chemical structures of top-twelve PCL compounds and their natural product-likeness scores are depicted in Fig. 4. The important feature of our method is that our predicted quasi-biogenic compound library includes chiral molecules, which are important characteristics in natural products. The previous reported methods were not able to generate chiral isomers [14, 17–19]. Top-200 PCL compounds and their natural product-likeness scores were listed in Additional file 3.

### The physico-chemical properties/descriptors profile of the predicted library

**Fig. 5:**
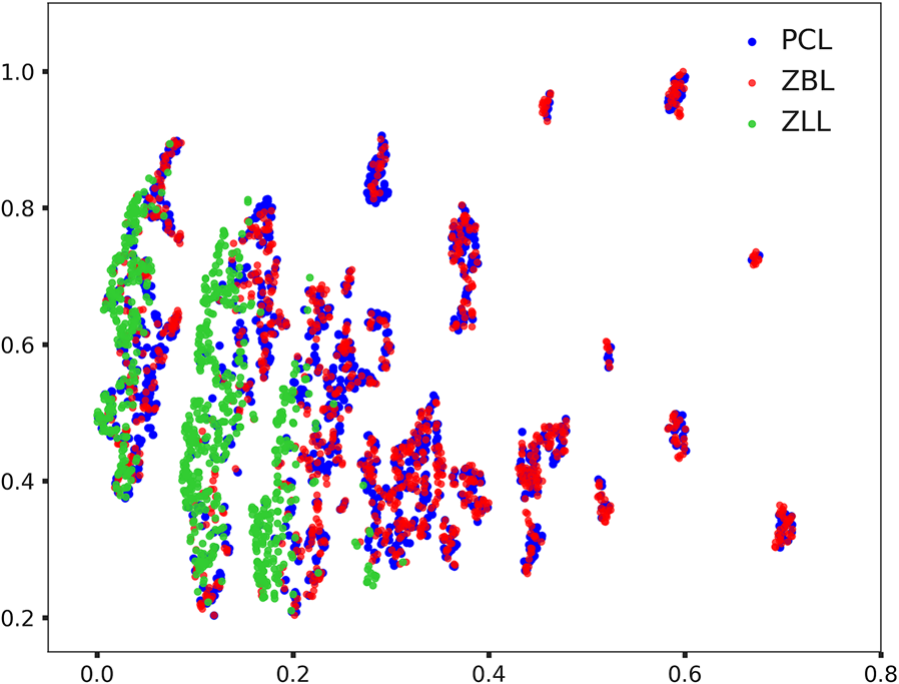
Two-dimensional t-distributed stochastic neighbor embedding (t-SNE) plot for PCL, ZBL, and ZLL. PCL and ZBL occupy almost the same chemical diversity space. ZLL partially occupies the chemical diversity space occupied by PCL and ZBL

A t-SNE plot was derived based on physico-chemical properties/descriptors (cLogP, MW, HDs, HAs, rotatable bonds, number of aromatic ring systems, and TPSA) to profile compound libraries, and compare their chemical diversity space occupations (Fig. 5). Again, PCL and ZBL occupied almost the same chemical diversity space. ZLL, however, only partially occupies the chemical diversity space occupied by PCL and ZBL. The plot also indicated that ZLL were not structurally as diverse as PCL and ZBL.

### Ability to reproduce biogenic molecules

Five-fold cross validation experiments indicated that the RNN model was mature after being trained for 20 epochs. The criterion of the training end was determined according to the change of the loss values during training. At this stage, quasi-biogenic molecules were sampled for studying the ability to reproduce already existed biogenic molecules. The results were represented in Table 1. Five-fold cross validation experiments were repeated for three times. The results demonstrated that the model can predict more than 25% compounds existing in the test library (TL). The RNN was robust because there were little fluctuations in three validation experiments as indicated at the last column of Table 1. It is worth noting that the RPP would slightly grow with longer training, even though the loss values were stable. To prevent overfitting of the model, we chose a moderate stage (20 epochs) for later experiments. The Epochs-Loss and Epochs-RPP curves were shown in Additional file 2.

**Table 1:** The reproducibility studies with five-fold cross validation experiments

Exp. no.	ZBL	TRL	TL	PCL	RP	RPP (%)
EXP1	153,733	122,896	30,747	153,733	7935 ± 267	25.81 ± 0.87
EXP2	153,733	122,896	30,747	153,733	7961 ± 341	25.89 ± 1.10
EXP3	153,733	122,896	30,747	153,733	7800 ± 120	25.37 ± 0.39

*ZBL* ZINC biogenic library; *TRL* training library; *TL* test library; *PCL* predicted compound library; *RP* repeated molecules existing in TL; *RPP* percent of repeated molecules (RP/TL)

**Fig. 6:**
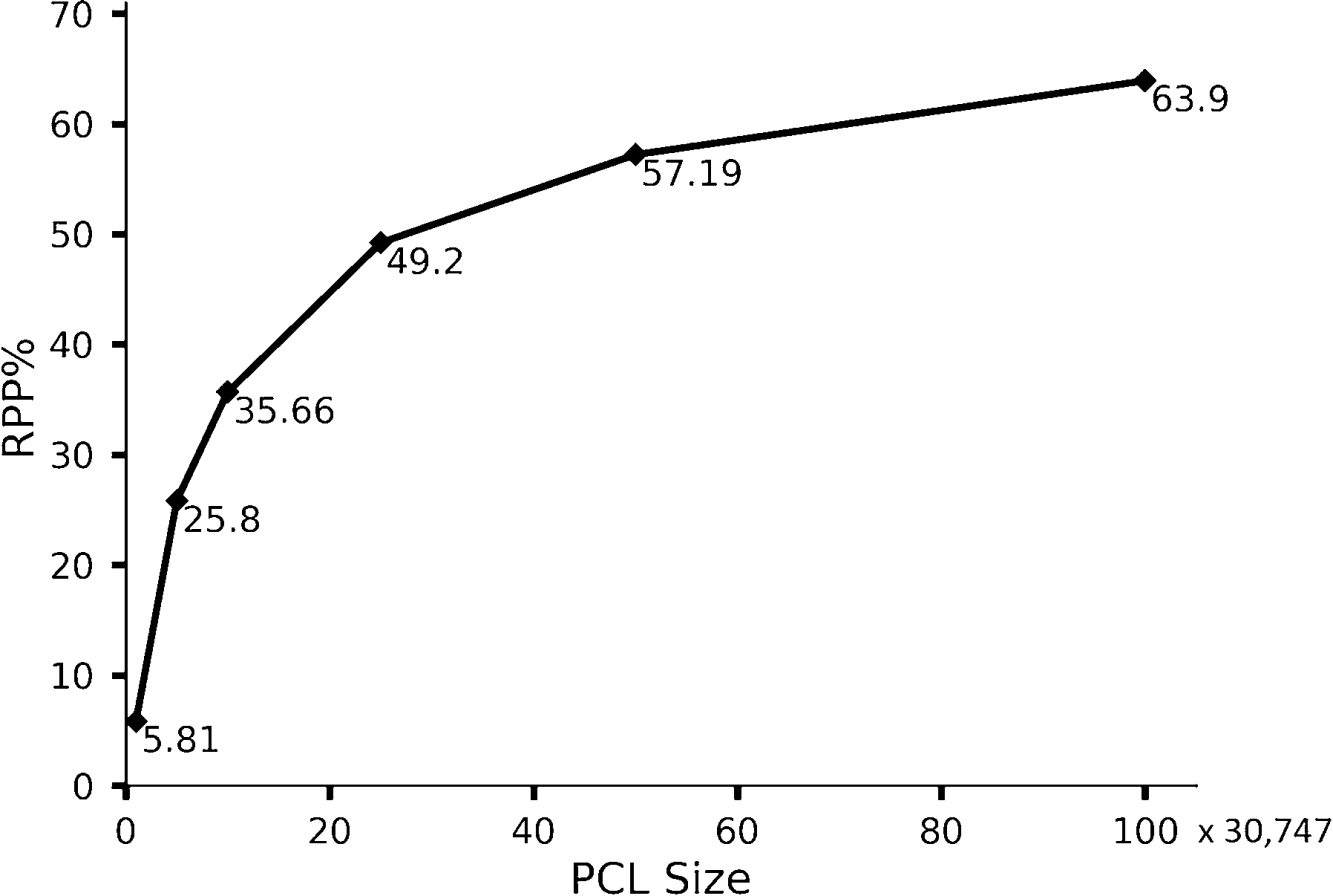
Reproducing known biogenic molecules in TL (30747) with different scale of generated set (1, 5, 10, 25, 50 and 100 times to TL)

At the first trial of the first five-fold cross validation experiment, we also generated a series of libraries with increased sizes (the 1, 5, 10, 25, 50 and 100 times of TL size, which is 30,747). As shown in Fig. 6, RPP increases exponentially when the PCL size grows to 30 × TL. And, RPP trends to be mature when PCL size increases further, and ends around 60% ~ 70%.

**Fig. 7:**
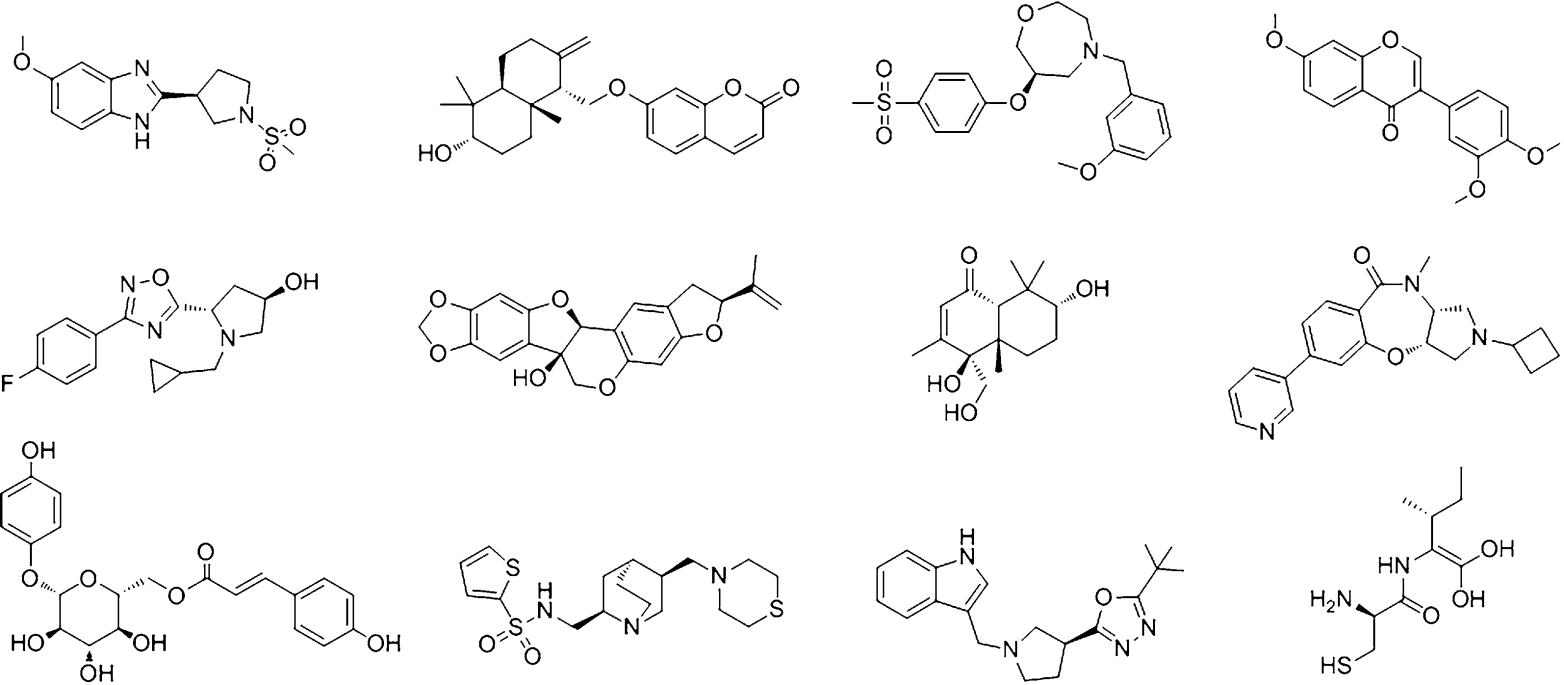
Chemical structures reproduced by the RNN model from TL

Several chemical structures reproduced by the RNN model from TL are listed in Fig. 7. More such compounds can be found in Additional file 4.

### Scaffold diversity and novelty of the predicted library

**Fig. 8:**
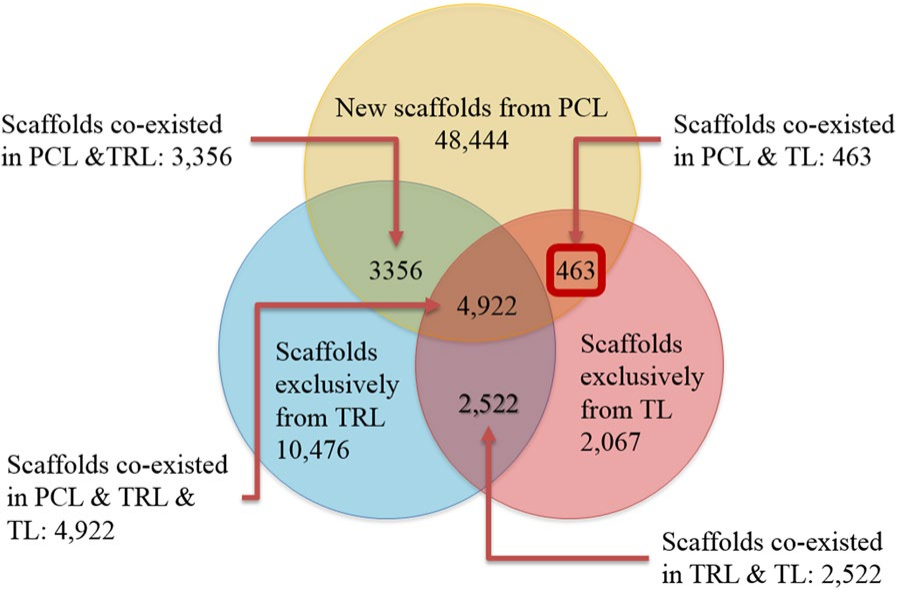
Scaffold diversity and novelty of the predicted compound library

At the first trial of the first five-fold cross validation experiment, the scaffolds of compound libraries TRL (122,896 compounds), TL (30,747 compounds), and PCL (153,733 compounds) were analyzed with scaffold-based classification approach (SCA). The results are depicted in Fig. 8. 48,444 new scaffolds are derived from PCL, which are 2 times more than the total scaffolds (23,806) derived from TRL and TL. 463 scaffolds are exclusively derived from both PCL and TL, indicating that the RNN model can generate new scaffolds, but predict repeated scaffolds in TL, which are outside the training library (TRL). To summarize, the RNN model is capable of generating diversified and novel compounds.

**Fig. 9:**
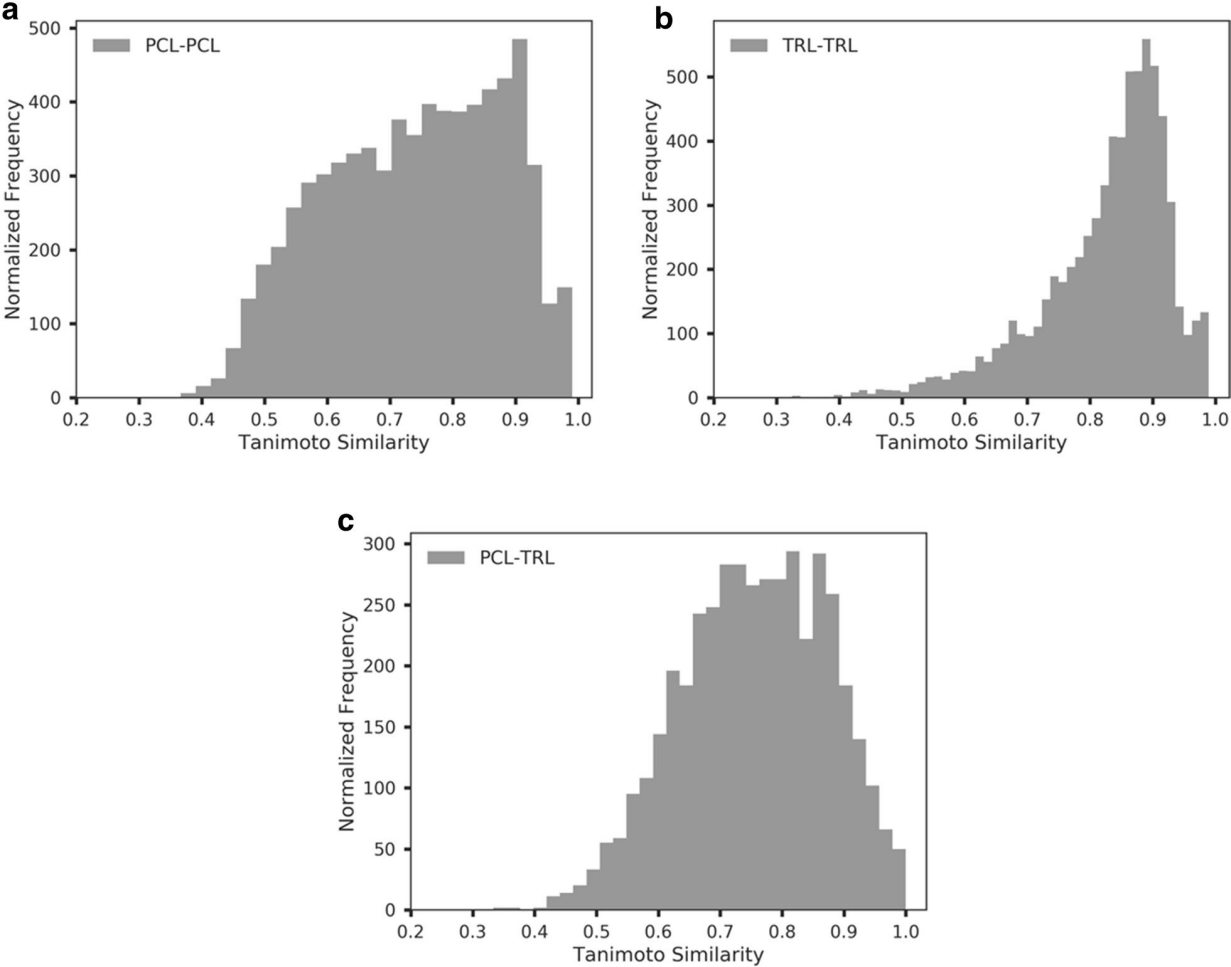
The nearest-neighbor scaffold similarity distributions of **a** PCL-TRL, **b** PCL–PCL and **c** TRL–TRL

Another way to measure the structural diversity and novelty of PCL is to check the distribution of the similarity of PCL and TRL. For each scaffold in PCL, we selected the most similar scaffold in TRL through calculating their Tanimoto similarity. The PCL-TRL similarity distribution was depicted in Fig. 9a, which demonstrates an unbalanced Gaussian distribution biased to higher similarity scores. The similarity values range between 50 and 100%. This implied that PCL scaffolds were similar to TRL scaffolds with variations in chemical diversity. We also calculated the nearest-neighbor Tanimoto similarity distributions of the scaffolds of PCL and TRL, which were depicted in Fig. 9b, c. The distributions of Tanimoto similarity indicated that the chemical space of PCL was diverse than TRL. This analysis further proved that the RNN model can generate diversified and novel quasi-biogenic compounds.

Some similar compound scaffold pairs between PCL and TRL were listed in Table 2.

**Table 2:** Six similar compound scaffold pairs between TRL and PCL

No.	TRL Scaffold	PCL Scaffold	Tanimoto similarity
1			0.94
2			0.87
3			0.82
4			0.80
5			0.77
6			0.71

### Potential bioactivities of the predicted library

For each PCL containing 150 K compounds, there were about 1% (1510 ± 221, mean ± SD) existed in the ChEMBL library, which are associated with bioactivities. Among those generated bioactive compounds, about 25% compound (371 ± 71) were found in the corresponding test libraries. Top-six such compounds and their activities were listed in Table 3.

**Table 3:** The generated six most bioactive molecules. 1, 2 and 3 existed in test set

No.	Structure	ChEMBL ID	IC_50_(nM)	Targets
1		CHEMBL65	0.004–1.4	P388; Plasmodium falciparum; CCRF-CEM; Jurkat;
2		CHEMBL489140	2–10	PBMC
3		CHEMBL2420226	~ 7	PBMC
4		CHEMBL815	2.25–7	Prostanoid FP receptor
5		CHEMBL2042018	5.2–6.2	Neurokinin 1 receptor
6		CHEMBL226036	9.2	Human herpesvirus 4

### Transfer learning for chemotype-biased library generation

Coumarin scaffold broadly exists in Rutaceae and Umbelliferae families. Its derivatives have many bioactivities such as activities of anticancer and anti-inflammatory [40–42]. The previously trained RNN model was re-trained with 2237 biogenic coumarin derivatives from ZBL. The model predicted 50 K compounds at 20, 50, or 100 epochs, respectively. In the three batches of the 50 K compounds, the compounds existing in TRL were excluded. 14,192 coumarin derivatives from ChEMBL23 database were extracted as bioactive reference library (BRL), in which the compounds duplicated in ZBL were removed. As a comparison, we also trained biogenic coumarin derivatives without transfer learning and followed the same processes described above. The scaffolds of each generated library were calculated with SCA for analyzing the diversity of chemical space.

The results of the transfer learning for chemotype-biased library generation were listed in Table 4. Comparing with the pre-trained RNN model, the number of coumarin derivatives is significantly increased (from 662 to more than 32 K). Besides, results demonstrated that the model without transfer learning generated compounds libraries with limited structural diversity and low correlation of bioactivity, though it can generate more coumarin derivatives. Also, when the number of transfer training epochs increased, the RNN model with transfer learning generated more coumarin-biased compounds. Table 4 also indicated that the number of coumarin-biased compounds trends mature along with the transfer epochs. The number of epochs should be limited to avoid overfitting.

**Table 4:** Results of transfer learning for chemotype-biased library generation

Model type	Epoch	TRL	BRL	PCL	Coumarin derivatives	PCL Scaffold	RP
Pre-trained	50	153,733(ZBL)	14,192	50,000	662	18,446	19
Direct-GRU	20	2237	14,192	50,000	37,647	6828	0
	50	2237	14,192	50,000	43,402	7642	0
	100	2237	14,192	50,000	43,094	7231	0
Transfer-GRU	20	2237	14,192	50,000	32,025	13,543	381
	50	2237	14,192	50,000	35,890	14,251	391
	100	2237	14,192	50,000	35,972	13,892	384

*TRL* training library; *BRL* bioactive reference library; *PCL* predicted compound library; *RP* number of compounds existing both PCL and BRL

The top-six predicted coumarin derivatives that existing in BRL and their bioactivities were listed in Table 5.

**Table 5:** The top-six predicted coumarin derivatives that existing in BRL and their bioactivities

No.	Structure	ChEMBL ID	IC_50_(nM)	Targets
1		CHEMBL307341	0.0006–0.0346	Monoamine oxidase B Monoamine oxidase A
2		CHEMBL177697	0.29	Influenza A virus
3		CHEMBL1078838	0.32	Influenza A virus
4		CHEMBL262328	0.68	HCT-116
5		CHEMBL465326	1.4	Liver microsomes
6		CHEMBL52229	3.1	P388

## Conclusions

In this work, for the first time, the gated recurrent unit deep neural network learning approach is applied in quasi-biogenic compound generation. We have also shown that a compound library biased on a specific chemotype/scaffold can be generated by re-training the RNN model through transfer learning with a focused training library.

In summary, our method is able to (1) generate libraries including stereochemistry, (2) significantly repeat compounds containing known bioactive compounds outside of the training sets, (3) create a de novo approach to generate focused libraries biased on a specified scaffold.

Our RNN model predicts biogenic compounds with a number of epochs depending on the size of the training data set. For a training set of about 150 K molecules, the number of training epochs can be less than 50, the optimized epochs can be figured out by monitoring the loss values and the capacity of generating new quasi-biogenic scaffolds. For a predicted biogenic compound, the average number of SMILES tokens is about 60 (similar to the one for a compound in the training set).

QBMG can be used to generate virtual biogenic compound libraries for pharmaceutical lead identification, and design focused library for lead optimization.

## Additional files

**Additional file 1.** GRU operations.

**Additional file 2.** Learning curves of biogenic library training.

**Additional file 3.** Top-200 PCL compounds and their natural product-likeness scores.

**Additional file 4.** Compounds reproduced by the RNN model from test library.

## Abbreviations

RNN: recurrent neural network
GRU: gate recurrent unit
ZBL: ZINC biogenic library
ZLL: ZINC biogenic-like library
PCL: predicted compound library
