# Supplementary material for: QBMG: quasi-biogenic molecule generator with deep recurrent neural network
Source: J Cheminform. 2019 Jan 17;11:5. doi: 10.1186/s13321-019-0328-9 (PMC6689867; doi:10.1186/s13321-019-0328-9)
Supplement: Supplementary file 1 — Additional file 1. GRU operations. [file 13321_2019_328_MOESM1_ESM.docx]

**Additional file 1：**

The GRU RNN model is presented in the form:

$h_{t}=\left( 1-z_{t} \right)\odot h_{t-1}+z_{t}\odot\tilde{h_{t}}$ (1)

$\tilde{h_{t}}=tanh\left( W_{h}x_{t}+U_{h}{(r}_{t}\odot h_{t-1} \right)+b_{h})$ (2)

$h_{t}$ is the hidden state at time $t$, $\tilde{h_{t}}$ the candidate hidden state, $x_{t}$ the input matrix, $W$ and $U$ are the weights of GRU cell and $b$ is the biases. The update and reset gates are presented as:

$z_{t}=sigmoid\left( W_{z}x_{t}+U_{z}h_{t-1}+b_{z} \right)$ (3)

$r_{t}=sigmoid\left( W_{r}x_{t}+U_{r}h_{t-1}+b_{r} \right)$ (4)
