# Supplementary material for: QBMG: quasi-biogenic molecule generator with deep recurrent neural network
Source: J Cheminform. 2019 Jan 17;11:5. doi: 10.1186/s13321-019-0328-9 (PMC6689867; doi:10.1186/s13321-019-0328-9)
Supplement: Supplementary file 2 — Additional file 2. Learning curves of biogenic library training. [file 13321_2019_328_MOESM2_ESM.docx]

**
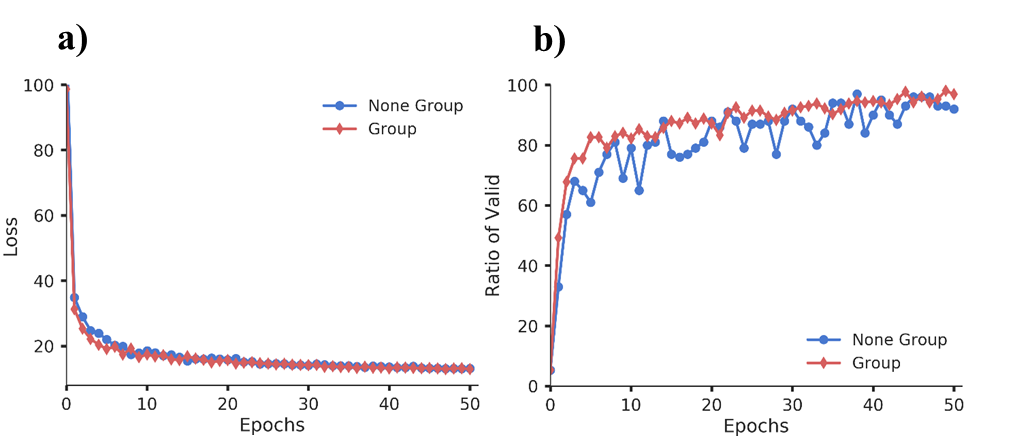
Additional File 2:**

**Fig 1.** Learning curves of biogenic library training with two different vocabulary construction methods mentioned in the work. (a) Epochs - Loss curve and (b) Epochs - Ratio of valid SMILES curve. ‘None Group’ is the conventional method. ‘Group’ is the vocabulary construction method used in this work. Model was trained on whole biogenic library.


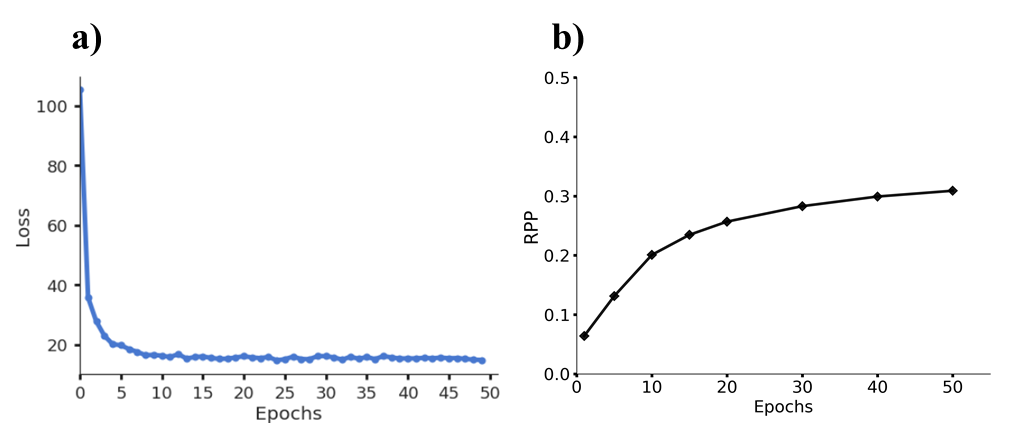


**Fig 2.** Example of Epochs-Loss and Epochs-RPP curves in five-fold cross validation experiments. After 20 epochs, longer training still slightly increases the repetition rate, even though the loss values become stable. Model was trained on 80% of biogenic library.
